# Intradural spine tumor surgery

**DOI:** 10.3171/2023.7.FOCVID23104

**Published:** 2023-10-01

**Authors:** Michelle J. Clarke, Paul McCormick, Mark Bilsky, Praveen Mummaneni

**Affiliations:** 1 Department of Neurological Surgery, Mayo Clinic, Rochester, Minnesota;; 2 Department of Neurological Surgery, Columbia University Irving Medical Center, New York, New York;; 3 Department of Neurological Surgery, Memorial Sloan Kettering Cancer Center, New York, New York; and; 4 Department of Neurological Surgery, University of California, San Francisco, California

## INTRODUCTION

Intradural spine tumors pose unique challenges to neurosurgeons because of their delicate location within or adjacent to the spinal cord and surrounding neural structures. The evolving field of intradural surgery has witnessed remarkable advances, including the innovative use of emerging technologies. In this issue of *Neurosurgical Focus: Video*, we are honored to feature contributions from leading experts, who share their knowledge to improve surgical outcomes, reduce morbidity, and enhance patient quality of life.

Each video presents a unique case, highlighting surgical challenges and the diversity of pathologies encountered. This collection of intradural spine tumor surgery videos showcases both classic and novel techniques to treat these diverse lesions. Demonstrated surgical techniques include microsurgical resection, endoscopic techniques, and minimally invasive surgeries, providing a comprehensive overview of the state-of-the-art practices in this specialized area.

A variety of extramedullary tumors are explored in this issue. Schwannomas are addressed through minimally invasive techniques in the videos from Deora et al. and Harary et al., while Bell and colleagues explore the resection of multiple tumors in the same procedure and Wilkinson et al. tackle a challenging extraspinal mass. Videos of challenging intradural extramedullary tumor resections include ventral meningiomas, presented by Rahmanov et al. and Rahman and colleagues; technical nuances in challenging meningioma cases, presented by Fisher and colleagues and Garufi et al.; and mixed intradural extramedullary pathologies in the videos from Macki et al. and Yunga Tigre and colleagues. Bhandari et al. present an impressive myxopapillary ependymoma resection in their video.

The challenges of intramedullary masses are showcased in multiple videos. Di Costanzo and colleagues demonstrate a pediatric epidermoid resection, and Erginoglu et al. present a hemangioblastoma with deterioration in a pregnant patient. Subependemoma resections are presented by Vignolles-Jeong et al., and Perez and colleagues demonstrate a resection with case review. Finally, Laws, Scherschinski, Bannach, Aaronson, and Kim and their colleagues present videos demonstrating the resection of ependymomas. Dauleac et al. explore the use of tractography to optimize intramedullary tumor resection while avoiding neurological deficits.

The videos in this issue explore decision-making, surgical techniques, and novel applications of technology, as well as provide technical pearls while serving as a historical record of the current state of intradural spine tumor surgery. We hope that this issue will be a valuable resource for neurosurgeons, residents, and medical professionals interested in intradural tumor surgery.

## Disclosures

Dr. Bilsky reported royalties from Globus Medical and DePuy Synthes Spine (PEEK carbon fiber cage), outside the submitted work. Dr. Mummaneni reported personal fees from DePuy Synthes, Globus, NuVasive, Stryker, BK Medical, Brainlab, and SI Bone; book royalties from Thieme Publishing and Springer Publishing; grants from AO Spine, NIH/NIAMS (U19AR076737), PCORI, Pacira, NREF, ISSG, and SLIP II; and stock ownership in Spinicity/ISD, outside the submitted work.

